# Revegetation on abandoned salt ponds relieves the seasonal fluctuation of soil microbiomes

**DOI:** 10.1186/s12864-019-5875-y

**Published:** 2019-06-11

**Authors:** Huyen-Trang Tran, Hao-Chu Wang, Tsai-Wen Hsu, Rakesh Sarkar, Chao-Li Huang, Tzen-Yuh Chiang

**Affiliations:** 1 Department of Life Sciences, National Cheng Kung University, Tainan, Taiwan 70101; 2 Department of Biology, Institute of Natural Science Education, Vinh University, Vinh, Nghe An 461010 Vietnam; 3 Taiwan Endemic Species Research Institute, Nantou, Taiwan 55244; 4 Institute of Tropical Plant Sciences, National Cheng Kung University, Tainan, Taiwan 70101

**Keywords:** Metagenomics, Salt pond restoration, Plant-microbe interaction, Seasonal fluctuation, Mangrove revegetation

## Abstract

**Background:**

Salt pond restoration aims to recover the environmental damages that accumulated over the long history of salt production. Of the restoration strategies, phytoremediation that utilizes salt-tolerant plants and soil microorganisms to reduce the salt concentrations is believed to be environmentally-friendly. However, little is known about the change of bacterial community during salt pond restoration in the context of phytoremediation. In the present study, we used 16S metagenomics to compare seasonal changes of bacterial communities between the revegetated and barren salterns at Sicao, Taiwan.

**Results:**

In both saltern types, Proteobacteria, Planctomycetes, Chloroflexi, and Bacteroidetes were predominant at the phylum level. In the revegetated salterns, the soil microbiomes displayed high species diversities and underwent a stepwise transition across seasons. In the barren salterns, the soil microbiomes fluctuated greatly, indicating that mangroves tended to stabilize the soil microorganism communities over the succession. Bacteria in the order *Halanaerobiaceae* and archaea in the family *Halobacteriaceae* that were adapted to high salinity exclusively occurred in the barren salterns. Among the 441 persistent operational taxonomic units detected in the revegetated salterns, 387 (87.5%) were present as transient species in the barren salterns. Only 32 persistent bacteria were exclusively detected in the revegetated salterns. Possibly, salt-tolerant plants provided shelters for those new colonizers.

**Conclusions:**

The collective data indicate that revegetation tended to stabilize the microbiome across seasons and enriched the microbial diversity in the salterns, especially species of Planctomycetes and Acidobacteria.

**Electronic supplementary material:**

The online version of this article (10.1186/s12864-019-5875-y) contains supplementary material, which is available to authorized users.

## Background

Ecological restoration and management have drawn the attention of conservation ecologists over the past decades [1]. Among the practices of ecological restoration, marine coastal restoration has received considerable attention [2]. Many coastal wetlands were ruined as a result of the development of salt pond industries. When these salt ponds are no longer needed they are abandoned. The landscape is seriously disturbed and natural habitats are lost. To recover these ecological losses, an increasing number of salt pond restoration projects have been undertaken over last two decades globally [3, 4].

Among the strategies for salt pond restoration, cultivation of mangroves is an environmentally-friendly approach [5, 6]. Artificial revegetation greatly decreases water and chemical usage in the soil improvement programs [7]. Mangroves improve the soil properties in saline environments shortly after their planting by increasing nutrient contents, enhancing water permeability, and reducing soluble salts [8–12]. The root systems secrete a wide diversity of root exudates that recruit various soil microorganisms [13]. Soil microorganisms are beneficial to the growth and health of plants, and help in stress alleviation, disease suppression, and nutrient acquisition [14–17]. Several microorganisms are able to promote plant growth in high salinity, including *Planococcus maritimus* CSSR02*, Bacillus pumilus*, and *Fusarium culmorum* FcRed1 [18–20]. Bacteria and fungi may help mangroves to adapt to the high salinity [7, 21]. Most of the beneficial effects are attributed to an interaction of the whole microbial community instead of any single species [22–25]. Therefore, understanding the interactions between soil microbiomes and mangroves in the salt pond restoration would provide insights into the application of revegetation in the ecological recovery.

The salt industry has been established in Taiwan since the past 300 years, with numerous salt ponds located along the southwest coast [4]. At Sicao in southern Taiwan ponds for salt treatments were established in 1919 and abandoned in 1996 [26]. The simultaneous restoration of salt ponds and preservation of the traditional method of salt production as a tourist attraction have involved the remediation of these abandoned ponds in the setting of an ecological park consisting of revegetated salterns and several barren salt ponds. Sicao has been selected as one of the “wetlands of international importance in Taiwan”, because it is a major breeding site of the black-winged stilt. A previous survey strongly correlated the faunal composition with mineral concentrations, particularly with salinity [27]. The salt concentration has remained high, fluctuating from 1.32–6.42% across seasons (http://wetland-tw.tcd.gov.tw/WetLandWeb/_download.php?id=2909&flag=2). Salt-tolerant plants have been introduced to the salterns to expedite habitat restoration. These revegetated plants are typically mangroves, including *Avicennia marina* (Forsk.) Vierh, *Kandelia candel* (L.) Druce, *Lumnitzera racemosa* Willd., and *Rhizophora mucronata* Lam [26]. The contrasting landscapes provide an opportunity to explore the effects of replanted mangroves on the saltern microbiome.

We compared the soil microbiomes between revegetated and barren salterns at Sicao. In addition, as the compositions of microbial community are not constant across seasons [28–31], sampling was made in different seasons to monitor the dynamics of saltern microbiome. The study addressed the following questions:

1. Did the revegetation of mangroves change the microbial composition and the seasonal dynamics in the abandoned salterns?
2. What is the microbiome assembly in the barren salterns?
3. Are there any microbes associated with the revegetation? If yes, what are their roles?
4. From the view of soil microbiome, how did the mangroves contribute to the restoration of salt ponds at Sicao?

## Results

### Changes of microbial communities across seasons

**Fig. 1:**
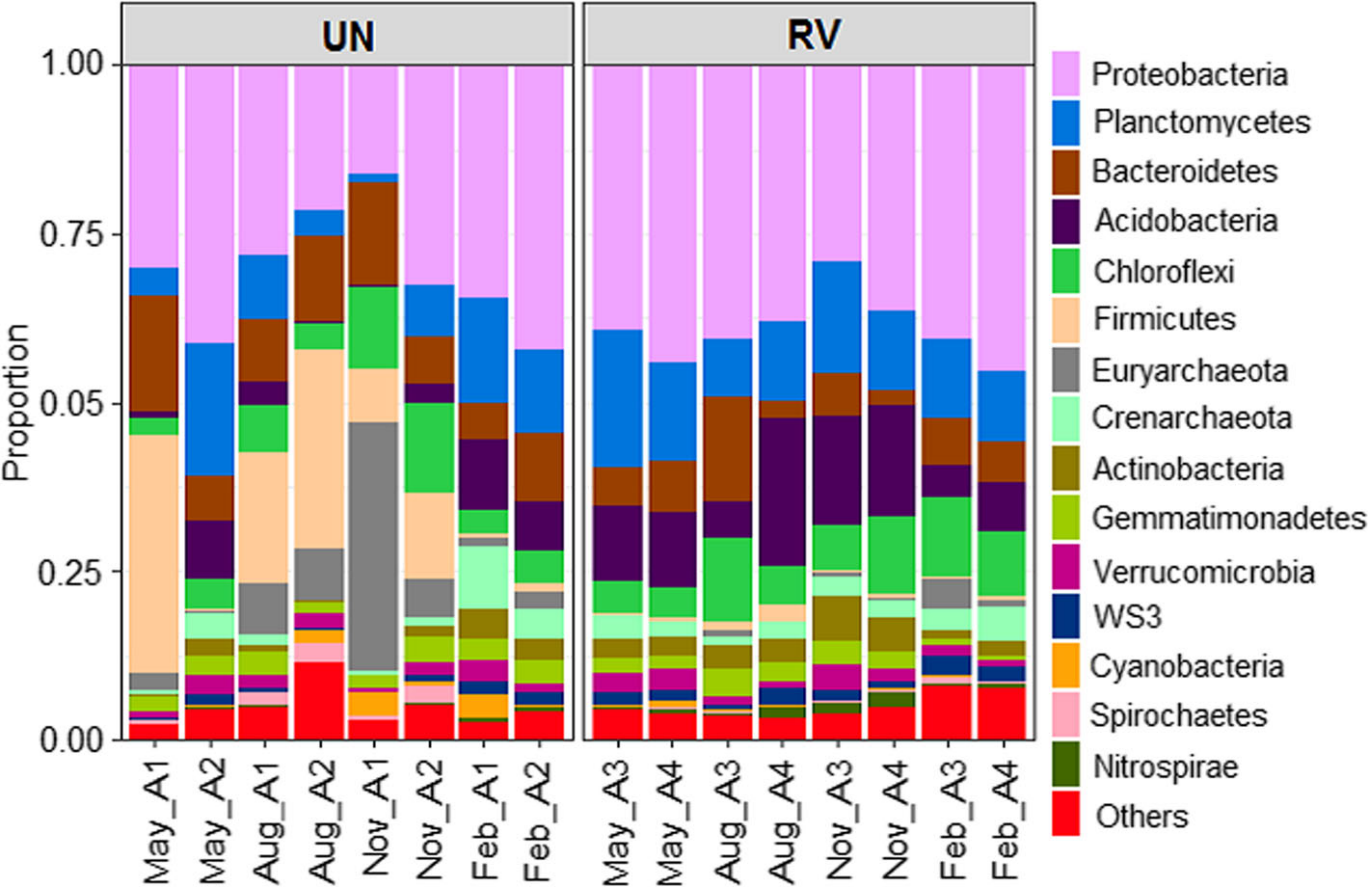
Read distribution of sequences assigned to the phylum level in UN and RV across seasons. The taxa of low abundances (< 1%) are pooled as ‘Others’

The summaries of reads and distribution of operational taxonomic units (OTUs) in each sample are provided in Additional files 1 and 2. In total, 2,134,601 PE sequences were recovered from the raw data. After excluding singleton haplotypes, 1,131,078 (52.99%) PE reads remained. Of these reads, 60.4–68.1% were mapped to Greengenes OTUs. Of the 8085 OTUs that were detected, 55.7–96.5% were assigned to bacteria, 2.2–33.8% to archaea, 0.19–10.4% to chloroplasts, and 0.0–0.2% to mitochondria (see Additional file 1). After rarefaction to 8375 reads, 6760 microbial OTUs remained. These OTUs were assigned to 66 phyla (99.9% reads), 157 classes (97.3% reads), 252 orders (85.2% reads), 283 families (59.6% reads), 426 genera (22.5% reads), and 148 species (2.3% reads). The bacterial community predominated in the Sicao samples, with abundance ranging from 62.4 to 97.8% (average = 92.5%), while archaea ranged from 2.3 to 37.6% (average = 7.5%). Archaea tended to decrease from untreated (UN) to revegetated (RV), while bacteria tended to increase in RV (Mann-Whitney, *p* = 0.015; see Additional file 3). At the phylum level, Proteobacteria, Planctomycetes, Chloroflexi, and Bacteroidetes were predominant both in UN and RV salt ponds across seasons (Fig. 1). Firmicutes and Euryarchaeota were present with a significantly higher abundance in UN compared with RV (Mann-Whitney, *p* < 0.02; see Additional file 3). Firmicutes were prevalent in UN from May to November and markedly decreased in February, while Euryarchaeota were prevalent in UN from August to November. In contrast, Acidobacteria and Actinobacteria were more abundant with the presence of salt-tolerant plants (respective average was 11.75 and 3.57%) than those without vegetation (4.31 and 1.68%, respectively, Mann-Whitney, *p* < 0.015; see Additional file 3). In addition, the top five most abundant OTUs in UN were assigned to *Halanaerobium* (8.15% with two OTUs), *Nitrosopumilus* (1.7%, belonging to Archaea), Planctomycetes (1.5%) and *Rhodobacteraceae* (1.3%). The top five most abundant OTUs in RV were identified as members of Betaproteobacteria (2.8% with two OTUs), *Piscirickettsiaceae* (1.2%), *Nitrosopumilus* (1.1%) and *Pirellulaceae* (1.1%) (see Additional file 4).

**Fig. 2:**
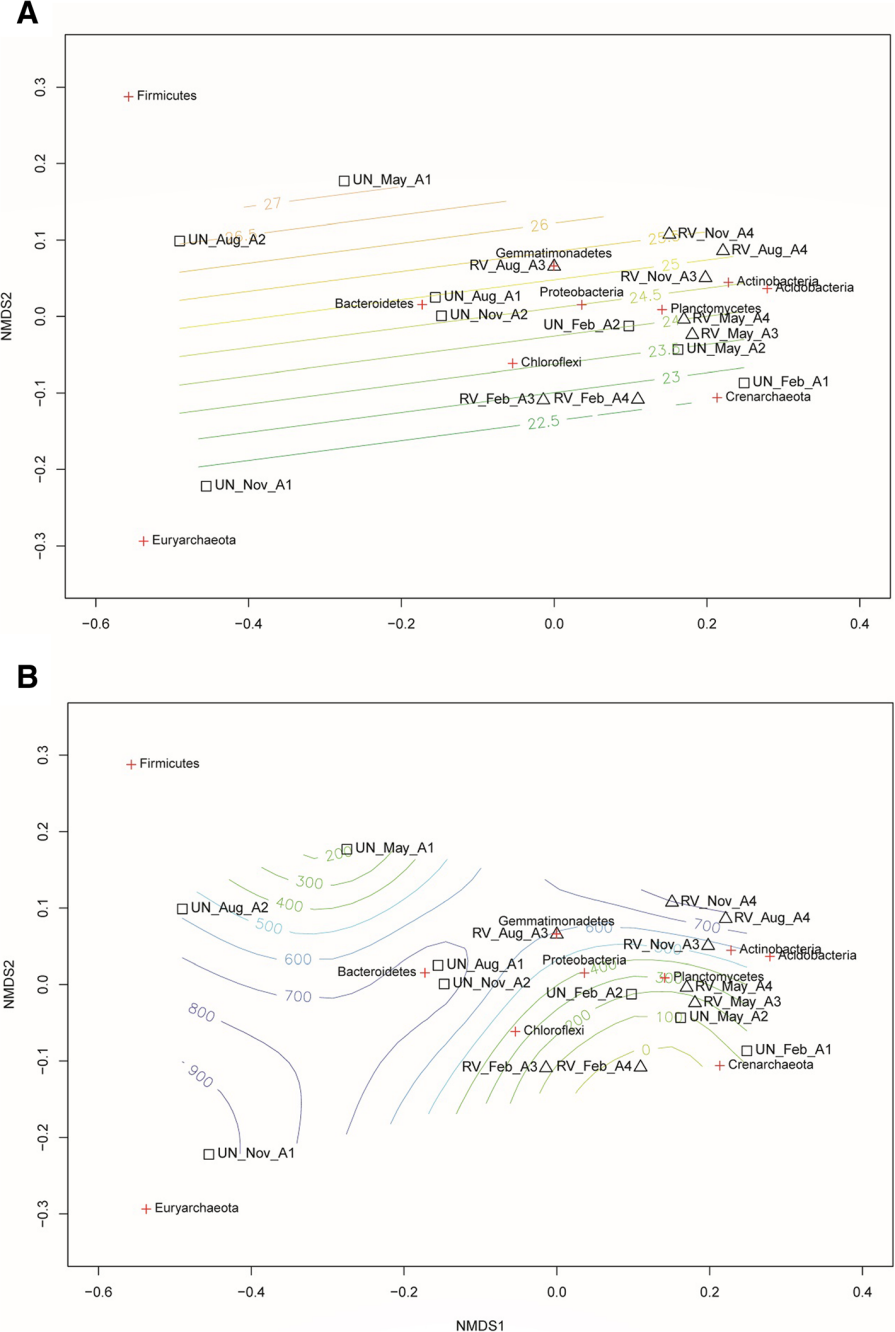
Non-metric multidimensional scaling (NMDS) plots with fitted environmental gradients of temperature (**a**) and accumulated precipitation (**b**). NMDS plots were generated with Bray-Curtis distance (stress 0.08). Square and triangle respectively indicate untreated (UN) and revegetated (RV). Gradient colors represent temperature (**a**) or accumulated precipitation (**b**). The distribution of the top 10 phyla are indicated by red crosses

We next used non-metric multidimensional scaling (NMDS) to visualize the differences in community composition (Bray-Curtis dissimilarity) between UN and RV at the phylum (Fig. 2) and OTU levels (see Additional file 5). No significant difference in composition was evident between UN and RV. After fitting the data of temperature and accumulated precipitation before sampling (Fig. 2), RV samples clustered near the center, while UN showed a more scattered pattern. These results suggested a closer association of samples in the RV groups with each other than with UN samples. The RV community displayed a higher association with temperature and precipitation than the UN samples. In addition, the distribution of Firmicutes was likely associated with high temperatures and low precipitation, whereas Euryarchaeota tended to live in low temperature, high precipitation habitats. Pearman’s correlation testing revealed that only Euryachaeota showed a significantly positive correlation with accumulated precipitation (*p* = 0.02; see Additional file 6).

**Fig. 3:**
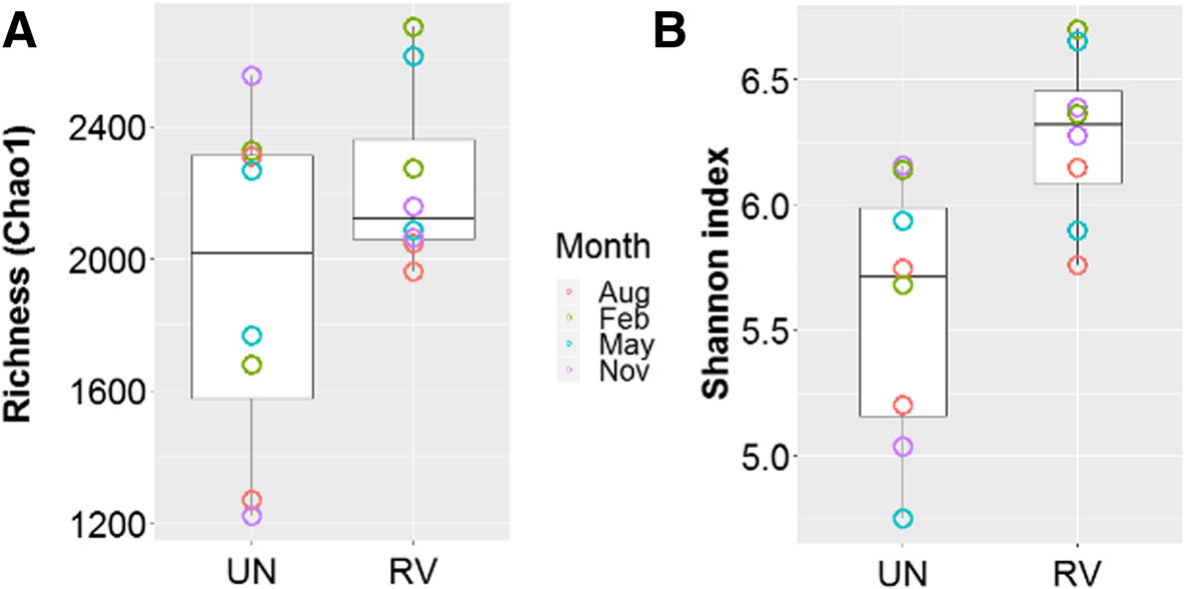
Comparision of species diversity between untreated (UN) and revegetated (RV) sites. Double asterisks indicate significant difference between UN and RV (Mann-Whitney test, *p* < 0.01). Each point is the Chao1 richness (**a**) or Shannon index (**b**) for the site at one sampling time

**Fig. 4:**
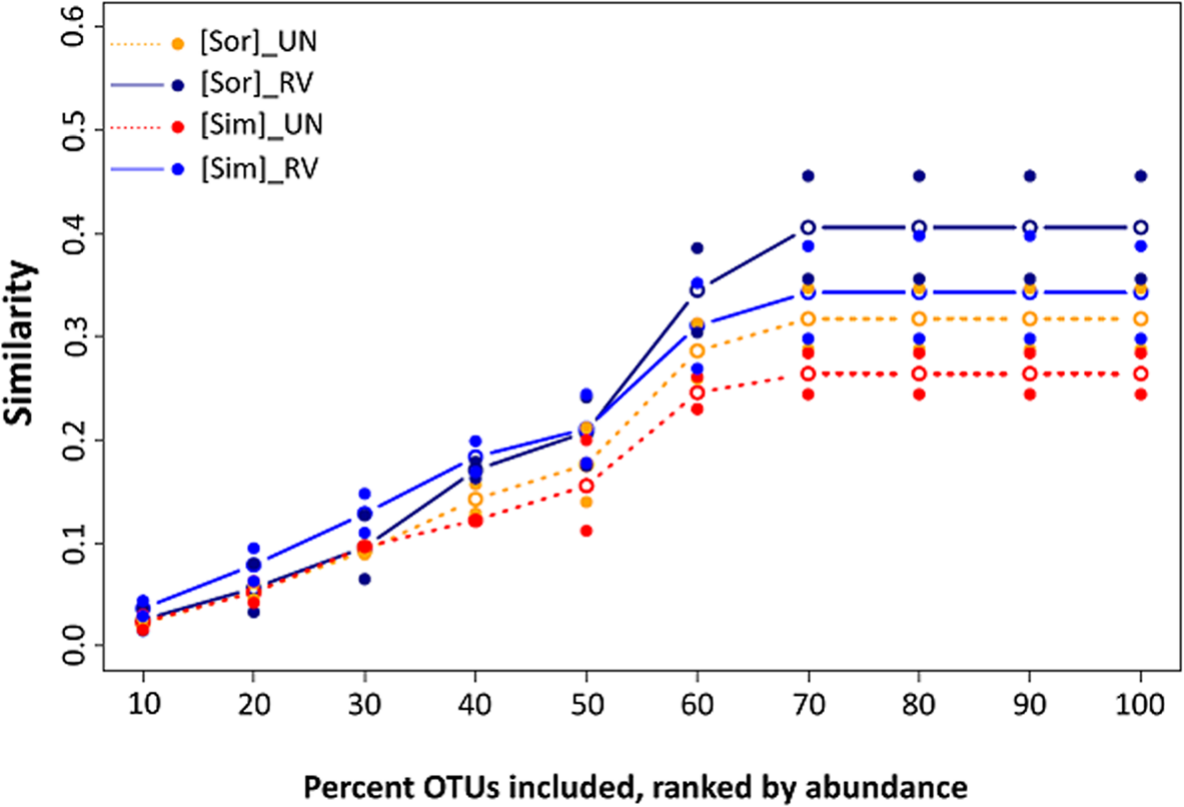
Partition of similarity at different levels of prevalence. Measured as Sørenson’s similarity ([Sør], orange and dark blue), and Simpson’s similarity ([Sim], red and blue). Each point is the multivariate community similarity calculated for a time series from one site. The analysis was repeated at 10 cutoff levels to remove less prevalent OTUs. The line is an average across two sites in one group

In addition, we calculated the OTU richness (Chao1), Shannon index (*H′*), Simpson similarity (Sim), and Sørenson’s similarity (Sør) for the saltern microbial communities across seasons. RV samples displayed no significant difference in OTU richness (Chao1) compared with UN (Mann-Whitney, *p* > 0.05), while the soil microbiota displayed higher *H′* (Mann-Whitney, *p* = 0.009; Fig. 3). From May 2013 to February 2014, the microbial diversity of UN tended to increase, while the trend was less obvious in RV. Both similarity indices plateaued when 70% of the OTUs (ranked by abundance) were included (Fig. 4). For Sør, the RV microbiome had a maximum value at 0.41 across seasons, and a lower value of 0.32 in UN. Likewise, Sim values were higher across seasons in RV than UN (0.34 vs. 0.26).

#### OTU persistency and prevalence

**Fig. 5:**
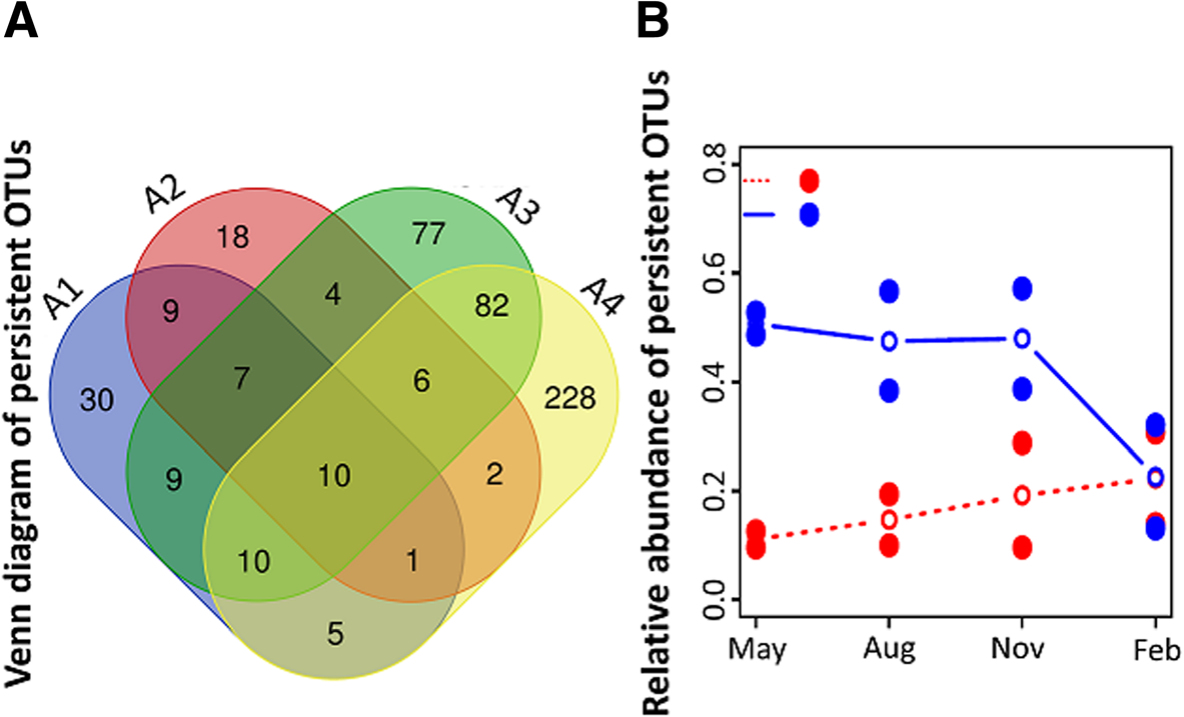
Characteristic of OTU persistence and prevalence at the four sites. **a** Venn diagram shows the unique and shared persistent OTUs between four sites. **b** Relative abundance of persistent OTUs in two groups. Each point is a relative abundance of persistent OTUs at each site in one month. The line is an average of the two sites in one group. Red and blue respectively indicate untreated (UN) and revegetated (RV)

The persistence of saltern microbiome was examined to confirm the observation of the increased microbial diversity and stability in the salterns following revegetation. In total, 498 persistent OTUs (7.4% of total OTUs) were detected from the four sampling sites. Of these, 81 (3.4%) and 57 (1.6%) were identified in sites A1 and A2 (UN), respectively, with 205 (5.5%) and 344 (10%) identified in sites A3 and A4 (RV), respectively (Fig. 5a). The observation of only 10 persistent OTUs in all sampling sites suggested that the seasonal persistency of soil bacteria fluctuated markedly in the salt evaporation ponds. The relative abundance of persistent OTUs also varied greatly across seasons. In UN, the persistent OTUs remained with low to moderate abundances of 10.8 to 22.8% across seasons. In RV, the prevalence was approximately 50% from May to November, but decreased dramatically to 22.6% in February, indicating contrasting trends between the presence and absence of salt-tolerant plants (Fig. 5b).

#### Local habitat preferences

**Fig. 6:**
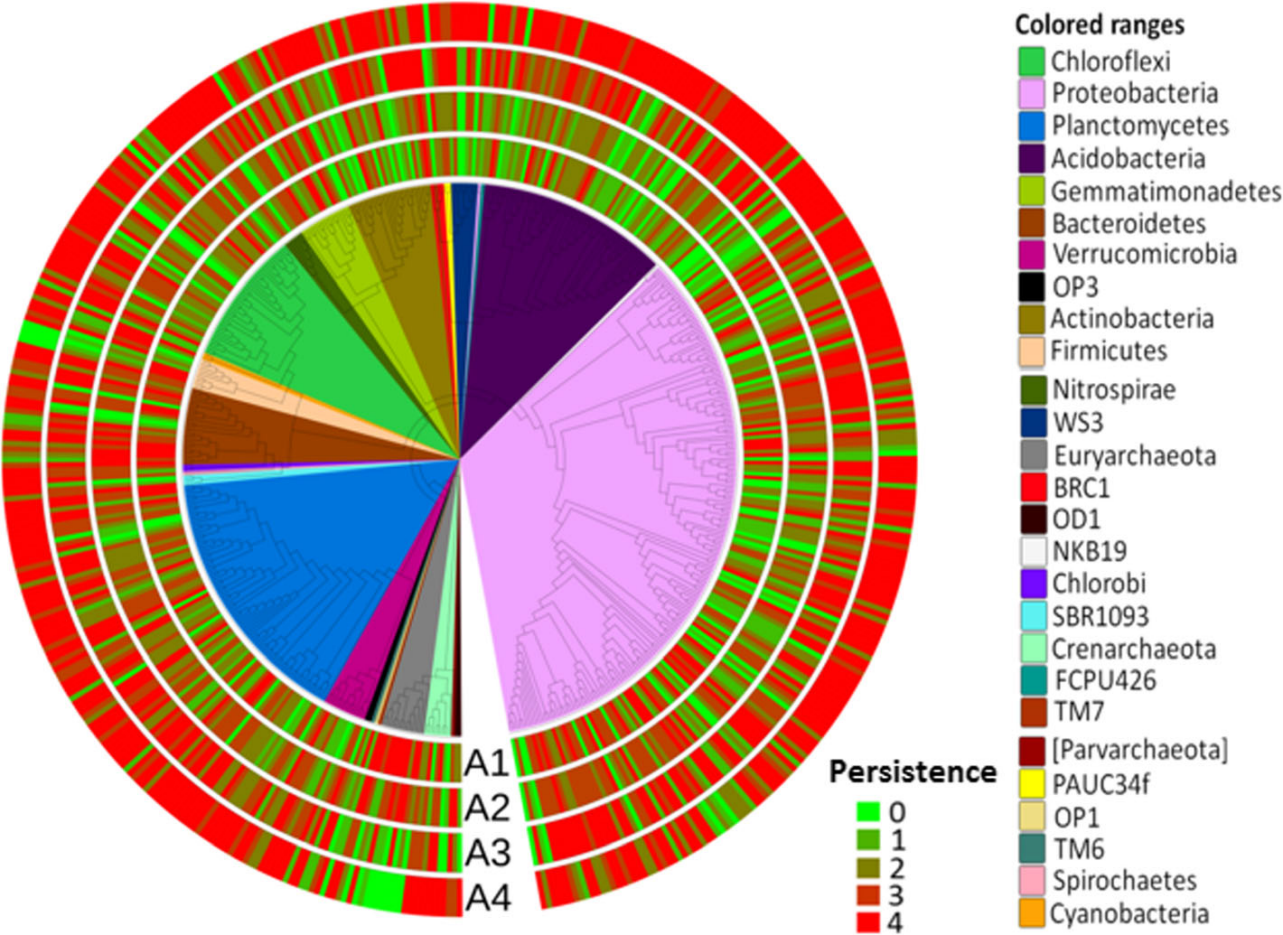
Taxonomy and occurrence of 498 persistent OTUs across each sample. The maximum likelihood tree of the bacterial domain was built based on the alignment of 498 persistent OTUs. Phyla are distinguished by branch color. The outer rings represent the status of OTU persistency in each site. Heat-map colors from green to red indicate for OTU occurrence of persistent OTUs across season in each site. The order is A1 to A4 from inside to outside

Clamtest analyses were performed to identify the specialists in UN and RV. Of the 498 persistent OTUs, 40 OTUs (8%) with extremely low frequencies were removed to avoid analytic noise. The remaining 329 OTUs were categorized as generalists (66.1%), while 33 (6.6%) and 96 (19.3%) were identified as specialists in UN and RV, respectively (see Additional file 7A). In general, most of the persistent OTUs belonged to Proteobacteria, Planctomycetes, and Acidobacteria (Fig. 6), and the relative abundance of the generalists was stable in the UN and RV groups (see Additional file 7B). There was only slight increase in the abundance in the RV group compared to the UN group (37.4 and 27.9%, respectively) (see Additional file 7B). The average relative abundance of specialist_UN and specialist_RV were 11.2 and 13.6%, respectively (see Additional file 7B). When salt-tolerant plants were present, 387 (87.5%) non-persistent OTUs in the barren salterns became persistent (Fig. 6). The average relative abundance of these OTUs was 16.4% in UN and 37.1% in RV (see Additional file 7C), suggesting that the prevalence also increased with the revegetation. These microorganisms mostly belonged to Proteobacteria, Acidobacteria, Planctomycetes, and Chloroflexi (see Additional file 7C). Among these phyla, Acidobacteria and Planctomycetes OTUs tended to be persistent after the revegetation (hypergeometric tests: FDR-adjusted *p*-values of 0.0003 and 0.04, respectively; see Additional file 7C). These results also supported the view that revegetation in abandoned salterns likely increased the persistency (stability) of soil microbiome.

### Metabolic pathways deduced by PICRUSt

**Fig. 7:**
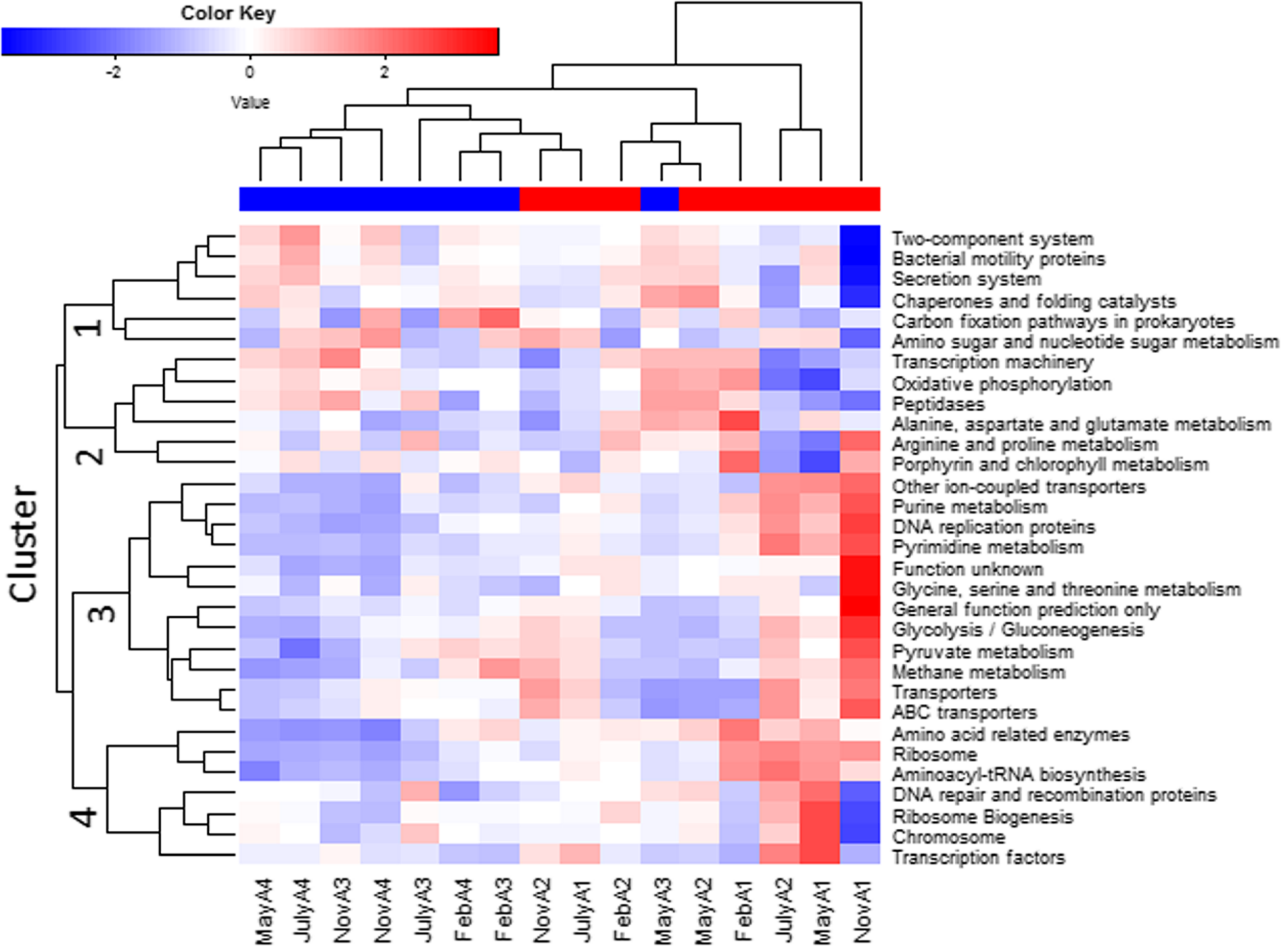
Heat-map visualization for functional pathways of 16 samples. The heat-map was generated based on a z-score transformation of top 30 functional pathways. The red and blue blocks below the dendrogram indicate the saltern types of UN and RV, respectively. The positive z-score are shown in red, and negative values are shown in blue

Using the PICRUSt program, 6909 pathways were detected in the Kyoto Encyclopedia of Genes and Genomes Orthology database. These comprised 316 level 3 pathways. Most of the pathways were found in both the saltern types, while six pathways were exclusively detected in RV with very low relative abundance (< 0.0001%). To simplify the result, the top 30 pathways were visualized using a heat-map analysis (Fig. 7). Most RV samples were clustered, except for MayA3, suggesting that the saltern types could be distinguished by metagenomic functions. The pathways could be grouped into four clusters. Of these, Cluster 1 showed higher abundance in RV samples. The cluster was composed of “Two-component system”, “Bacterial motility proteins”, “Secretion system”, “Chaperones and folding catalysts”, “Carbon fixation”, and “Amino sugar and nucleotide sugar metabolism”. Among them, the abundance of the “Two-component system” was significantly higher in RV than that in UN (Mann-Whitney, *p* = 0.02), implying some associations between this pathway and the revegetation.

## Discussion

Salt evaporation ponds have usually been established by removing the native mangroves or other salt-tolerant plants from the coastal wetlands. Inevitably, after the de-vegetation, many ecological functions of wetlands were deleteriously affected, as revealed by the reductions in the diversity of fish, birds, invertebrates, and microorganisms [32, 33]. Restoration of such damage by processes of natural succession usually requires centuries. To expedite habitat remediation, artificial revegetation involving the introduction of native plants is often practiced [34, 35]. The salt pond system at Sicao is a perfect example.

Evaluating soil health by investigating the microbial composition in the soil ecosystem has been suggested [36]. In the present study, metagenomic analyses revealed a microbial community with higher species diversity in the revegetated salterns (Fig. 3b). A likely scenario for the increased diversity is that the revegetation may have created novel niches for immigrants from neighboring fields, as indicated by the existence of unique microorganisms in the revegetated salterns (belonging mainly to Proteobacteria, Planctomyces, Bacteroidetes, and Acidobacteria; see Additional file 9). The metagenomic data indicated that over the course of succession following revegetation, 1639 and 2531 OTUs might have been lost and gained, respectively. The net difference of 896 OTUs was likely attributable to the habitat recovery with the presence of salt-tolerant plants. In addition, the increasing number of specialists in the revegetated salterns also suggested that more ecological niches were developed by phytoremediation. During the process, plants are able to release a wide array of root exudates that mediate various interactions with soil microorganisms, including an expansion of available carbon sources [37]. A trend of increased diversity was also evident for bacteria after the revegetation in terms of the species composition, especially those belonging to Acidobacteria, Actinobacteria, Proteobacteria, Chloroflexi, and Planctomycetes (see Additional file 3). Additionally, the nourishing effect by vegetation was more pronounced for bacteria, as the number of archaeal species decreased by 59 and the average relative abundance of archaea decreased from 11.0 to 4.1% after revegetation (see Additional file 3). This also indicates that many archaea may survive in severe environments. At the phylum level, the reduction of archaea was due to the loss of Euryarchaeota taxa, while Crenarchaeota were stable. The most prevalent Euryarchaeota in the Sicao salterns belonged to the *Halobacteriaceae*. They are highly halophilic, adapt to high osmotic pressure, and require over 100–150 g/L salt for survival [38]. Several salt-tolerant plants are able to take up excess ions from soil and relieve the interference to the roots via secretion or accumulation. For example, the mangrove *Luminitzera* can store excess salt in the succulent leaves, while *Avicennia* utilizes salt glands to eliminate the salts [39, 40]. Such mechanisms enable the plants to reduce the salinity in neighboring soil, as revealed by a previous study showing that the revegetation on saline soil reduces salinity up to 10-fold [41]. These results imply that the return of plants transforms the environment to a state that is detrimental to halophilic prokaryotes. Similar to Euryarchaeota, Firmicutes displayed a reduction following the revegetation. The most predominant Firmicutes in the barren salterns belonged to *Halanaerobiaceae*, a moderately halophilic family that thrives under high temperatures (34–40 °C) and salinity (approximately 10%) [42]. Accordingly, the decline in halophilic species resulted in the corresponding reductions in the abundance of Firmicutes and Euryarchaeota.

Firmicutes also displayed pronounced seasonal variation in the barren salterns. The relative abundance was higher in May and August (19.5 and 16.0%, respectively), lower in November (8.6%), and extremely low (< 1%) in February; this displayed a positive correlation between the relative abundance and temperatures (see Additional file 6). Higher temperatures may trigger salt tolerance of the microorganisms [43]. Accordingly, the low temperatures in February (< 20 °C) limited growth. Euryarchaeota also displayed a similar pattern; they were abundant in August and November, and less abundant in February and May. This is likely due to the accumulating precipitation (see Additional file 5). Collectively, in response to the climate changes, the microbial flora widely fluctuated in the saltern ecological systems.

Temperature and precipitation may have critical roles in changing the microbial composition across seasons [29–31, 44]. As mentioned above, Firmicutes might be influenced by temperature and Euryarchaeota by precipitation. In addition, the nature of the salterns (barren versus revegetated) could be influential (Fig. 2). The NMDS analysis revealed a correlation of the saltern microbiome with the accumulated precipitation. In the rainy months of August and November, the microbial communities of the barren and revegetated salterns were different and were located at the left and upper right regions of the NMDS representation, respectively. In the dry seasons, there was less of a difference between two saltern types, except for UN_May_A1, which clustered in the lower right region (Fig. 2b). This pattern suggested that the influence of vegetation is more pronounced during the rainy seasons. In Taiwan, periods of accentuated rainfall are coupled with severe weather, including typhoons and the ‘plum rains’ of late spring and early summer. These natural forces are expected to have great impacts on the composition of the soil microbiome [45], as revealed by the dynamics of saltern microflora in the rainy seasons. Interestingly, unlike the scattered pattern of the UN samples, the RV microbiome exhibited stepwise changes along an upward trajectory with the increasing accumulation of precipitation (Fig. 2b). This finding suggests that plants may have resulted in the stable transition of the soil microbiome across seasons, increasing the ecological stability of saltern environments. A similar community structure was also found in the cool and dry month of February, which likely reflected the stable climate in winter. The NMDS analysis further revealed a stable and stepwise transition of the microbiome in the revegetated salterns across seasons (Fig. 2). Sørenson and Simpson similarity indices also revealed a higher stability in the revegetated salterns, as the soil microbiome was less variable than that of the barren salterns (Fig. 4). Based on the the microbial composition (Figs. 5 and 6), the stable transition in revegetated salterns was likely associated with the increasing number of persistent microbes, as the persistence of species is an intuitive indicator of ecological stability [46]. In the revegetated salterns, 441 persistent microorganisms were detected, which was approximately 4-times the number of persistent microorganisms in the barren salterns (111 OTUs). Persistent microorganisms accounted for up to 60% of the soil microbiome after revegetation, while the barren salterns were predominated by non-persistent microorganisms across seasons. Apparently, ecological stability was strengthened by revegetation in the salterns. There are two possible contributors to the increase in the number of persistent microbes—new colonization events and the microbial conversion from non-persistent to persistent. More than 85% of the persistent microbes in the mangrove salterns were also detected as non-persistent microbes in the barren salterns, whereas < 10% of the persistent microbes were derived from colonization events. Most of the persistent microorganisms (78%) did not display preferences to barren or mangrove salterns. These results indicate that the colonization events may not be the main reason for the recruitment of persistent microbes. Alternatively, plants likely stabilized the resident soil microorganisms in the salterns by alleviating the influences of climate changes. Root exudates contain abundant photosynthetic metabolites, including simple sugars and amino acids [13, 47, 48]. The expansion of organic matter after revegetation in the saline soil has been reported [7, 41, 49]. During bioremediation, the increased available resources would provide better nourishment for soil microorganisms that had survived the unfavorable conditions [50]. This might be one of the reasons that existing microbes were more likely to become persistent upon the introduction of salt-tolerant plants. Contrastingly, the PICRUSt analysis suggested that the revegetated saltern is significantly enriched of the bacterial function of “Two-Component System” (TCS) (see Additional file 8). The TCS is composed of sensor and transducer components that can respond to various environmental changes [51]. The system links prokaryotic cells with their environments [52]. The genomic abundance of TCS is associated with trophic levels, as indicated by the greater number of TCS related genes in prokaryotic copiotrophs in comparison to oligotrophs [52]. As copiotrophs thrive in nutrient-rich environments, the metabolic analysis suggests that revegetation improves the growth condition for soil microorganisms.

Among the microbes that showed enhanced persistence after the revegetation, Acidobacteria and Planctomycetes were particularly favored by the presence of mangroves (see Additional file 7C). Acidobacteria are a common component of rhizosphere communities [53] and important contributors to the microbial assembly in aquatic and terrestrial ecosystems [54]. Acidobacteria are likely to possess active metabolisms that promote plant growth [55–57]. Plant diversity is in turn a critical factor in shaping their community structure, probably via providing carbon sources to attract specific Acidobacteria toward the root systems [58]. Likewise, the Planctomycetes are reportedly associated with mangroves and seagrasses [59–61]. Halophytes are able to alter the distribution of bacteria, especially those belonging to the Planctomycetes [62]. These bacteria are involved in the nitrogen cycle, including the anammox bacteria with anaerobic oxidation of soil ammonia in saline sediments [62]. On the other hand, Actinobacteria were also enriched in revegetated salterns, especially for the order Acidimicrobiales (see Additional files 3 and 10). Acidimicrobiales have been reported as predominant microbes in the rhizosphere of salt-tolerant plants, including *Agave*, *Halimione*, and *Sarcocornia* [63, 64]. However, as only a few species in this order have been isolated and studied [65], their interaction with plants still requires more research.

The collective findings indicate that revegetation is the beginning of the restoration toward a well-functioning wetland. The bacteria may be involved in the growth promotion of salt-tolerant plants as well as the processes of the nitrogen cycle.

## Conclusions

Salt-tolerant plants tend to stabilize the soil microbiome of the Sicao salt ponds. These plants may help the non-persistent microbes thrive during climate transition. As the number of persistent microbes increased, the ecological stability of mangrove salterns improved because of the increasing diversity of the soil microbiome. With revegetation, the numbers of Firmicutes and Euryarchaeota markedly declined, while Acidobacteria and Planctomycetes thrived across seasons. Such plant-microbe interactions may facilitate the succession processes in barren salterns and restore the ecological functions of a coastal wetland.

## Methods

### Site descriptions and sampling

This study was conducted in a national ecological park with several salt evaporation ponds at Sicao, Tainan, Taiwan (23.026°N, 120.141°E). Four experimental sites (A1-A4) were set. Two sites had been revegetated with salt-tolerant plants [*Kandelia candel* (L.) Druce, *Lumnitzera racemosa* Willd, *Rhizophora mucronata* Lam, and *Avicennia marina* (Forsk) Vierh, *Phragmites australis*]. The other two sites had been untreated and were used as controls (see Additional file 11)*.* Accordingly, the experimental sites were classified as untreated saltern (UN) and saltern with revegetation (RV).

In Tainan, the rainy season generally starts in April and ends in September (see Additional file 12). Accordingly, we collected soil samples from the saltern fields in May, August, November, and February in 2013 and 2014 to assess the microbial dynamics across rainy and dry seasons. Each sample was a homogeneous mixture of three sub-samples from the solid soil surface in the same site (approximately 20 cm in depth). Soil samples were stored on ice and transported to the laboratory immediately. In total, 16 samples were obtained.

### DNA extraction and PCR

Soil DNA was isolated with the PowerSoil® DNA Isolation Kit (MO BIO Laboratories, Inc., USA). A 789F primer (5′-TAG ATA CCC SSG TAG TCC - 3′) and 1053R nucleotide reverse primer (5′- CTG ACG RCR GCC ATG C-3′) were used to amplify the target V4-V6 region of 16S-rRNA. The primer pair was chosen because of its wide coverage of bacterial lineages [66, 67]. For each sample, PCR was performed in eight to 12 tubes. The reaction volume was 50 μL. Each reaction volume consisted of 1 × *Taq* 2x Master Mix Red (Ampliqon, Denmark), 0.1 μM of each primer, and 10 ng template DNA. PCR conditions were 95 °C for 5 min; 27 cycles of 95 °C for 30 s, 50 °C for 30 s,72 °C for 50 s, and a final extension at 72 °C for 10 min. PCR products were visualized using 1.2% agarose gel electrophoresis and purified using a Gel/PCR DNA extraction Kit (Geneaid, Taiwan). The purified PCR products were precipitated and concentrated with isopropanol. The DNA concentration was quantified using a Qubit® 2.0 Fluorometer (Invitrogen, USA).

### Metagenomic sequencing and analysis

#### OTU generation and rarefied OTU table

PCR products were sequenced using an Illumina MiSeq with 250-bp paired-end sequencing. All raw sequence data has been deposited in the GenBank Sequence Read Archive database (http://www.ncbi.nlm.nih.gov/sra/) under the accession number SRP134270. Using a Perl-scripted pipeline, raw reads were quality (trimmed) filtered (average quality value > = 20 and length > = 100 bp). After quality filtering, non-paired reads were discarded, and paired reads were assembled according to the overlapping sequences. The primer sequences were trimmed from the assembled sequences, haplotypes were generated by merging identical sequences, and the abundances were determined. Singleton haplotypes were discarded due to possible sequencing errors. Finally, OTUs were identified by clustering haplotypes with the OTUs of the Greengenes database (August 2013 version) at 97% sequence identity using the ‘pick_closed_reference_otu.py’ function implemented in the QIIME software package 1.9.0 [68]. Mitochondrion, chloroplast, and singleton OTUs were excluded before further analysis for bacteria and archaea. The “rrarefy” function in the *vegan* v.2.4–4 package [69] was used to rarefy all samples to 8375 reads (corresponding to the lowest number of microbial reads detected in NovA3). All community analyses were based on the rarefied results.

#### Community analysis

The *vegan* package was also used to estimate OTUs richness (Chao1), Shannon index, Simpson similarity, Sørenson similarity, and NMDS (meta-MDS function) based on Bray-Curtis distance. The “ggscatter” function in the *ggpubr* package was used for Pearson correlation coefficient analysis between the abundance of Firmicutes and Euryarchaeota with temperature and accumulated precipitation before sampling [70]. The OTUs that occurred at all time points in a sampling site were defined as persistent OTUs, while the remainders were considered non-persistent OTUs. The distributions of persistent OTUs were visualized with an online Venn diagram tool (http://bioinformatics.psb.ugent.be/webtools/Venn/). Functional pathway profiles from 16S metagenomics were predicted by PICRUSt version 1.1.3. [71]. Heat-map for functional pathways was generated in R with a z-score transformation.

The clamtest function of the vegan package was used to classify specialist and generalist with and without the presence of replanted salt-tolerant plants. All parameters for clamtest were set as default values with the “supermajority” rule [72].To detect the phyla that were enriched in the group of persistent generalists, a hypergeometric test was used. The *p*-values were corrected with a false discovery rate of 0.05. A statistically significant result was one where the corrected p-value was < 0.05. A maximum likelihood tree based on 428 persistent generalist OTUs was generated by MEGA7 [73]. The tree was visualized with Interactive Tree Of Life v.3.0 [74].

## Additional files

Additional file 1: **Table S1.** Summary of sequencing reads from 789F-1053R marker. (XLSX 14 kb)

Additional file 2: **Table S2.** Summary of haplotypes and Operational Taxonomical Unit (OTU) numbers detected using 789F-1053R markers. (XLSX 15 kb)

Additional file 3: **Figure S1.** Box-plots of relative abundance of the two kingdoms and the top 10 phyla between UN and RV. Asterisks denote statistically significant difference evaluated by Mann-Whitney test between UN and RV. *, *p*-value <=0.05, and **, *p*-value< 0.01. (TIF 1657 kb)

Additional file 4: **Figure S2.** Distribution of taxon abundances among OTUs (with singletons removed) detected in (A) UN and (B) RV. Top five OTUs in relative abundance with taxa assignation in (C) UN and (D) RV. (TIFF 52 kb)

Additional file 5: **Figure S3.** NMDS (non-metric multidimensional scaling) plots of all 16 samples based on Bray-Curtis distance at OTUs level (stress 0.09). (TIFF 66 kb)

Additional file 6: **Figure S4.** Pearson correlation coefficient analysis of temperature and accumulated precipitation with (A) Firmicutes and (B) Euryarchaeota. Red and blue lines present linear regression of UN and RV samples, respectively. Red and blue shades denote for the confidence intervals at 95%. (TIFF 89 kb)

Additional file 7: **Figure S5.** Local habitat preferences. (A) Habitat preferences of the persistent OTUs. Based on the clamtest, each OTU was classified as specialists in UN (red squares), specialists in RV (green diamonds), “too rare” (blue triangles), or generalist (black circles). B). Distribution of habitat preference of the persistent OTUs in each site. C). Phylum distribution of the 387 OTUs that are persistent in RV but non-persistent in UN. The asterisks indicate significant enrichment of the number of persistent OTU for the phyla at RV (hypergeometric test, *p* = 0.04). (TIFF 137 kb)

Additional file 8: **Figure S6.** Abundance comparison for the six pathways of cluster 1 between UN and RV. The asterisk (*) *represents a statistical significance*. (TIFF 38 kb)

Additional file 9: **Figure S7.** Top five phyla of unique microorganisms in RV. (TIFF 25 kb)

Additional file 10: **Figure S8.** Relative abundance of order Acidimicrobiales in phylum Actinobacteria. A). OTUs number. B). Reads. (TIFF 31 kb)

Additional file 11: **Figure S9.** Information for sampling sites. Mapping of sampling was drawn based on the google map data 2018. Photos of sampling sites were provides along with sites. (TIFF 373 kb)

Additional file 12: **Figure S10.** Average monthly of (A) temperature and (B) precipitation of Tainan during 1990–2014. (TIFF 32 kb)

## Data Availability

The raw sequencing reads is deposited in GenBank’s Sequence Read Archive (SRA) database (http://www.ncbi.nlm.nih.gov/sra/) under the accession number SRP134270.

## Abbreviations

DNA: Deoxyribonucleic acid
NMDS: Non-metric multidimensional scaling
OTUs: Operational Taxonomic Unit
PCR: Polymerase chain reaction
PE: Paired-end sequencing
RV: The saltern with revegetation
SRA: Sequence Read Archive
UN: The untreated saltern
