# Supplementary figures and images for: Revegetation on abandoned salt ponds relieves the seasonal fluctuation of soil microbiomes
Source: BMC Genomics. 2019 Jun 11;20:478. doi: 10.1186/s12864-019-5875-y (PMC6558789; doi:10.1186/s12864-019-5875-y)

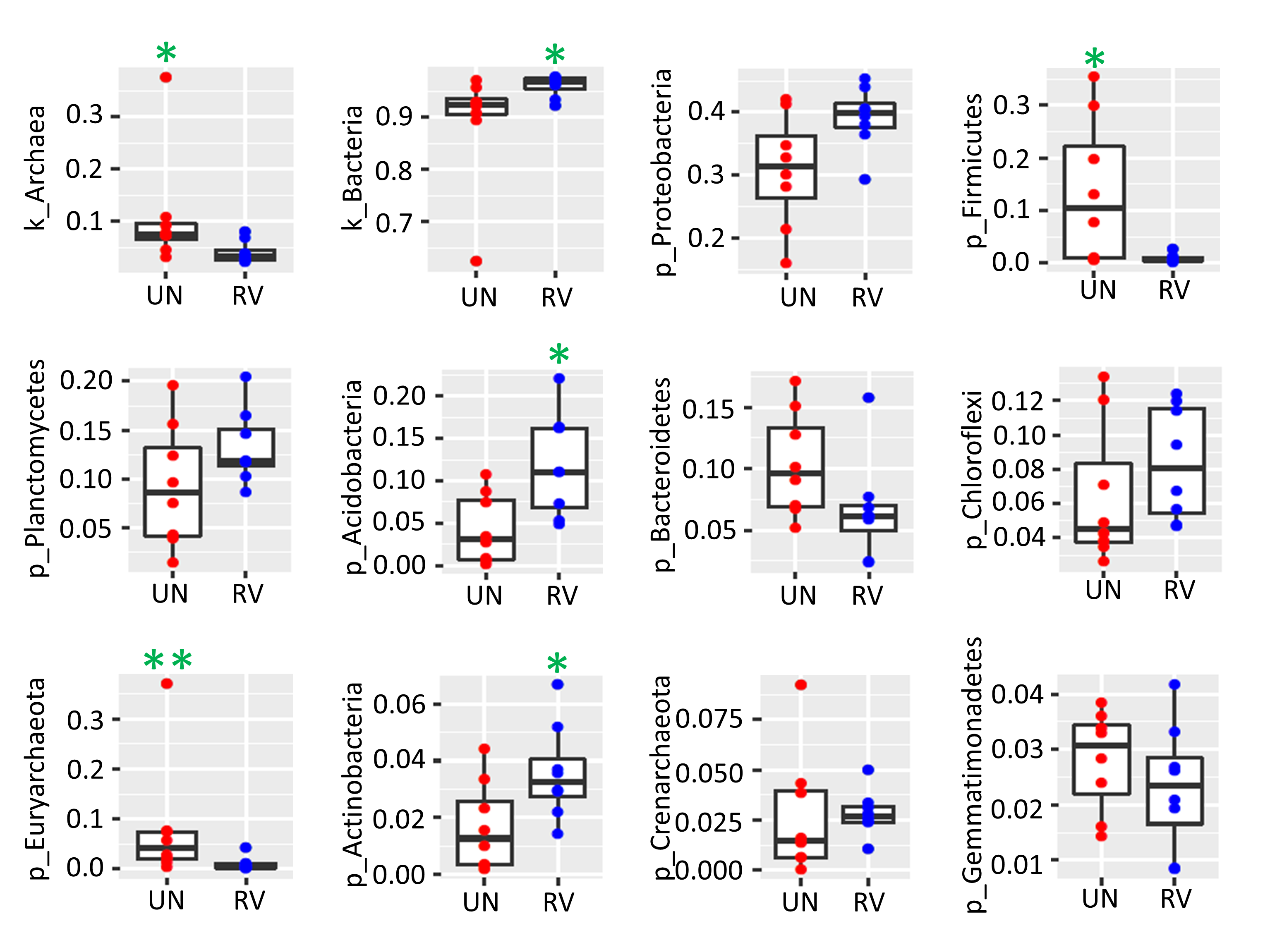

Supplement: Supplementary file 3 — Figure S1. Box-plots of relative abundance of the two kingdoms and the top 10 phyla between UN and RV. Asterisks denote statistically significant difference evaluated by Mann-Whitney test between UN and RV. *, p-value <=0.05, and **, p-value< 0.01. (TIF 1657 kb) [file 12864_2019_5875_MOESM3_ESM.tif]

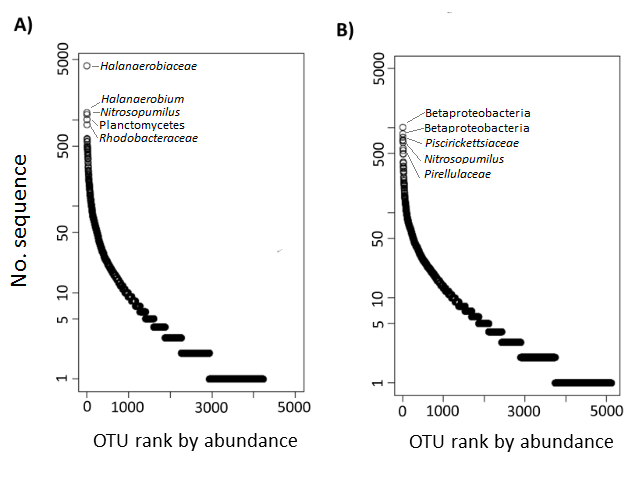

Supplement: Supplementary file 4 — Figure S2. Distribution of taxon abundances among OTUs (with singletons removed) detected in (A) UN and (B) RV. Top five OTUs in relative abundance with taxa assignation in (C) UN and (D) RV. (TIFF 52 kb) [file 12864_2019_5875_MOESM4_ESM.tiff]

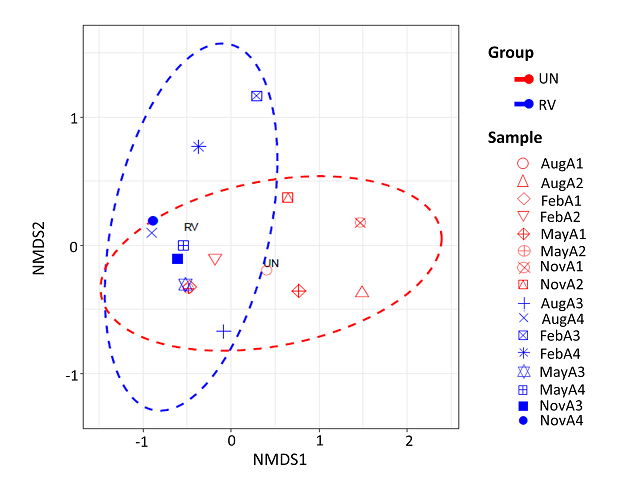

Supplement: Supplementary file 5 — Figure S3. NMDS (non-metric multidimensional scaling) plots of all 16 samples based on Bray-Curtis distance at OTUs level (stress 0.09). (TIFF 66 kb) [file 12864_2019_5875_MOESM5_ESM.tiff]

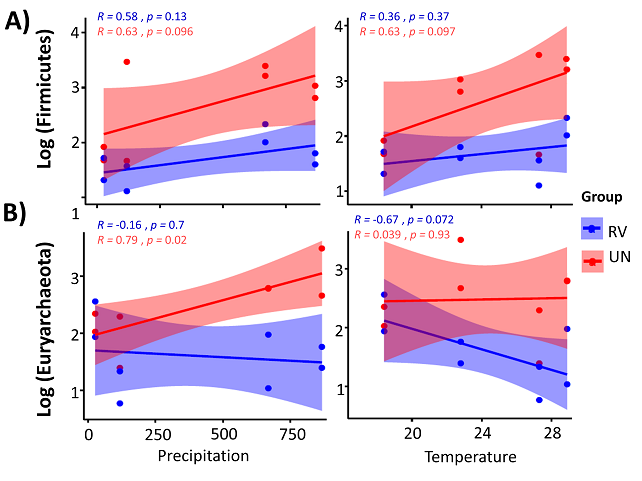

Supplement: Supplementary file 6 — Figure S4. Pearson correlation coefficient analysis of temperature and accumulated precipitation with (A) Firmicutes and (B) Euryarchaeota. Red and blue lines present linear regression of UN and RV samples, respectively. Red and blue shades denote for the confidence intervals at 95%. (TIFF 89 kb) [file 12864_2019_5875_MOESM6_ESM.tiff]

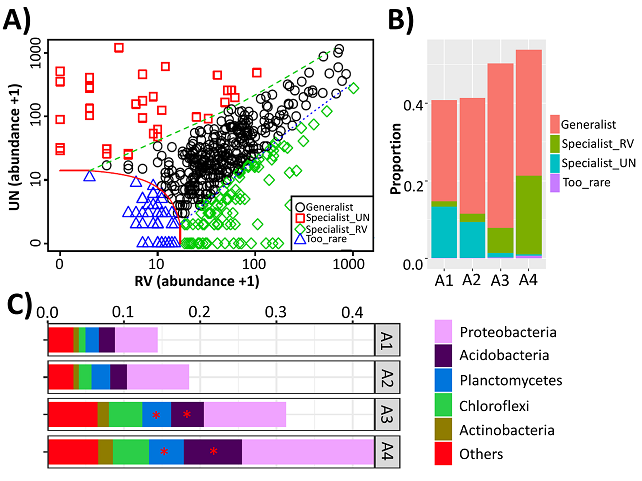

Supplement: Supplementary file 7 — Figure S5. Local habitat preferences. (A) Habitat preferences of the persistent OTUs. Based on the clamtest, each OTU was classified as specialists in UN (red squares), specialists in RV (green diamonds), “too rare” (blue triangles), or generalist (black circles). B). Distribution of habitat preference of the persistent OTUs in each site. C). Phylum distribution of the 387 OTUs that are persistent in RV but non-persistent in UN. The asterisks indicate significant enrichment of the number of persistent OTU for the phyla at RV (hypergeometric test, p = 0.04). (TIFF 137 kb) [file 12864_2019_5875_MOESM7_ESM.tiff]

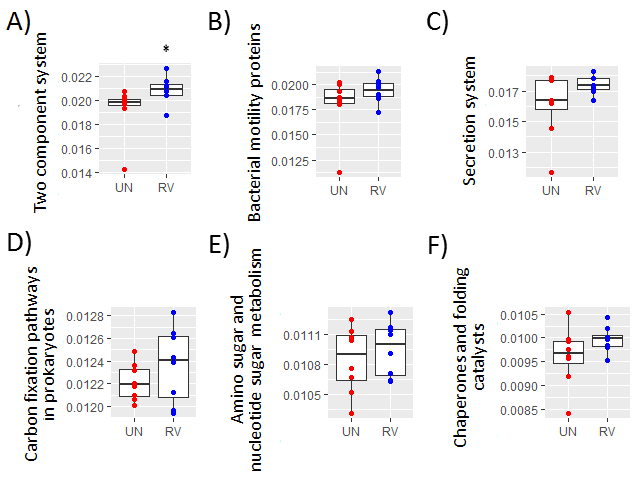

Supplement: Supplementary file 8 — Figure S6. Abundance comparison for the six pathways of cluster 1 between UN and RV. The asterisk (*) represents a statistical significance. (TIFF 38 kb) [file 12864_2019_5875_MOESM8_ESM.tiff]

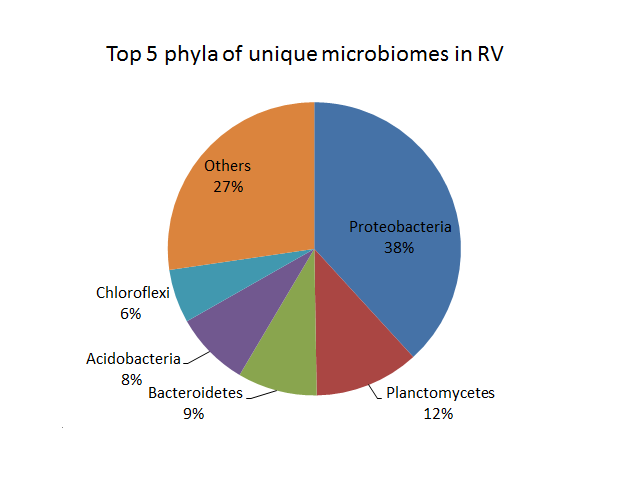

Supplement: Supplementary file 9 — Figure S7. Top five phyla of unique microorganisms in RV. (TIFF 25 kb) [file 12864_2019_5875_MOESM9_ESM.tiff]

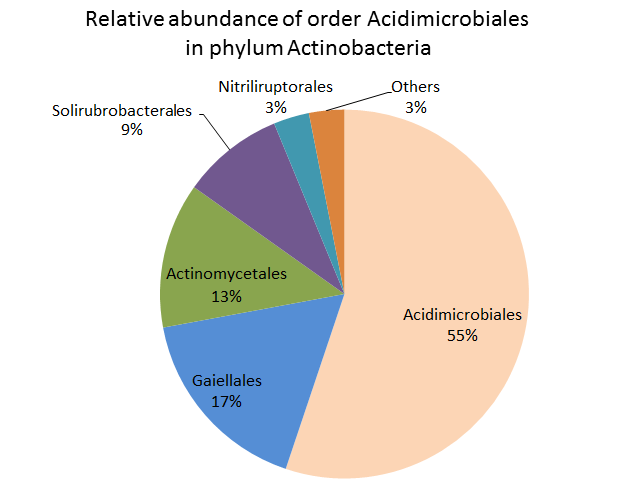

Supplement: Supplementary file 10 — Figure S8. Relative abundance of order Acidimicrobiales in phylum Actinobacteria. A). OTUs number. B). Reads. (TIFF 31 kb) [file 12864_2019_5875_MOESM10_ESM.tiff]

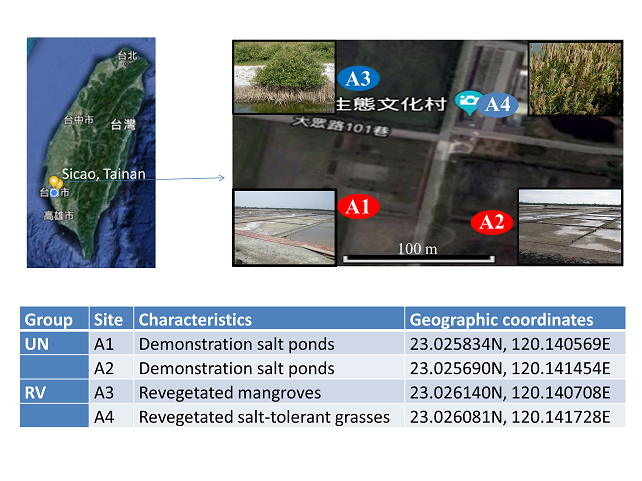

Supplement: Supplementary file 11 — Figure S9. Information for sampling sites. Mapping of sampling was drawn based on the google map data 2018. Photos of sampling sites were provides along with sites. (TIFF 373 kb) [file 12864_2019_5875_MOESM11_ESM.tiff]

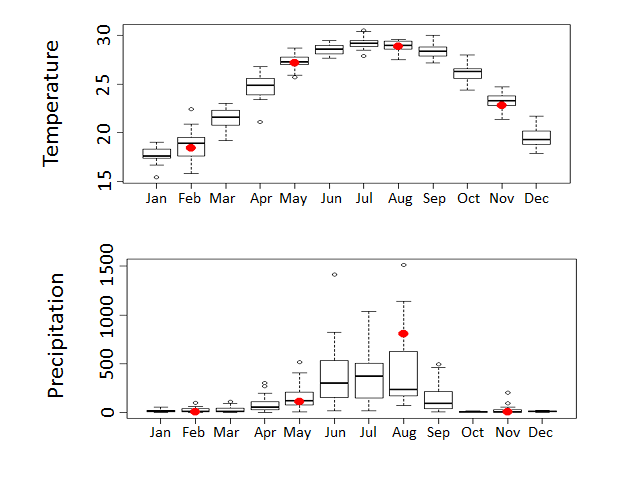

Supplement: Supplementary file 12 — Figure S10. Average monthly of (A) temperature and (B) precipitation of Tainan during 1990–2014. (TIFF 32 kb) [file 12864_2019_5875_MOESM12_ESM.tiff]
